# Psychosocial Impact of Sarcoma: Challenges and Adaptation, a Meta‐Synthesis

**DOI:** 10.1002/pon.70534

**Published:** 2026-07-07

**Authors:** R. Beghean, L. O'Driscoll, E. Ryan, P. Carty, F. Larkin, F. O'Keeffe

**Affiliations:** ^1^ University College Cork (UCC), School of Applied Psychology Cork Ireland; ^2^ St. Vincent's University Hospital (SVUH) Dublin Ireland

**Keywords:** adaptation, bone cancer, cancer, challenges, psychosocial impact, qualitative research, sarcoma, soft tissue sarcoma, thematic synthesis

## Abstract

**Objectives:**

Sarcoma is a rare and heterogeneous cancer frequently associated with significant psychosocial burden. This review aims to synthesise qualitative research to examine the psychosocial impact of sarcoma in adults, focusing on how individuals experience and make sense of disruption across diagnosis, treatment, recovery, and survivorship, the ways they adapt to these challenges, and the psychosocial needs they identify throughout the sarcoma trajectory.

**Methods:**

A preregistered systematic review (PROSPERO CRD42024571502) was conducted following PRISMA and ENTREQ guidance. Four databases were searched (PsycINFO, MEDLINE, CINAHL, and Web of Science), and peer‐reviewed qualitative studies involving adults with sarcoma were included. Updated search conducted on 30/12/2025. Study quality was appraised using CASP, and data were synthesised using inductive thematic synthesis to integrate experiential accounts and generate analytical insights.

**Results:**

Forty studies published between 2008 and 2025 involving 538 participants were included, with most rated high methodological quality. Five themes captured sarcoma as a cumulative psychosocial disruption, beginning with diagnostic delay and uncertainty, intensifying through invasive treatment and prolonged recovery, and extending into enduring changes in body image, function, identity, and social participation. Adaptation emerged as a dynamic, ongoing process involving self‐management, meaning‐making, selective information engagement, and reliance on relational and professional support. Persistent unmet needs were identified, particularly regarding specialist, coordinated care and sarcoma‐informed rehabilitation.

**Conclusions:**

Experiences of sarcoma as adults are characterised by prolonged psychosocial disruption requiring adaptation across all stages of the illness. Findings highlight the importance of specialist, continuous, and rehabilitation‐focused sarcoma care to support long‐term adjustment and wellbeing.

## 1 Background

Sarcoma is a rare heterogeneous group of malignant tumours arising in bone, cartilage, and soft tissue [1]. It accounts for approximately 1% of adult malignancies and includes more than 70 histological subtypes across diverse anatomical sites [2]. Sarcoma's rarity and clinical heterogeneity contribute to diagnostic delay, limited awareness, and disrupted care pathways, further compounding patients' distress [3, 4].

While representing a small proportion of cancers, sarcoma can be associated with disproportionate psychosocial burden relative to more common cancers [5, 6]. Individuals consistently report lower quality of life, elevated distress, anxiety, depression, and fear of recurrence [6, 7]. Functional impairment, altered body image, and social disruption are also common alongside unmet informational and psychosocial needs [8, 9]. Franzoi et al. [7] provided strong evidence of sarcoma's substantial psychosocial burden; however, that review, along with others, also highlighted major methodological limitations. Clinical heterogeneity, varied patient experiences, lack of disease‐specific tools, and inconsistent outcome measures constrain accurate assessment and interpretation of its impact [2, 10].

In recent years, there has been a rapid surge of qualitative studies exploring the lived experiences of sarcoma, illuminating the emotional and social dimensions of living with a rare cancer. Two recent meta‐syntheses have synthesised this literature. Meek and Baliousis [11] explored the psychological impact of sarcoma along the illness trajectory, identifying themes of uncertainty and identity change and comparing these experiences with those of people with Kaposi sarcoma. Wang et al. [12] described broader lived experiences and unmet needs, including stigma, financial strain, and social barriers.

However, several aspects of sarcoma experience remain less clearly understood. First, both reviews included wide age ranges spanning adolescence to older adulthood (13–85 years), potentially obscuring adult‐specific experiences as developmental stage can influence how people relate to illness [13]. Second, although existing evidence highlights both psychological and broader social challenges, these domains have largely been examined separately, leaving limited understanding of how emotional, social, relational, and functional disruptions interact in adults' lives. An adult‐focused synthesis that integrates these domains may therefore provide a more coherent psychosocial understanding of sarcoma. Finally, although coping and emotional responses have been described in previous syntheses [11, 12], less is known about how adults with sarcoma adapt to these wider psychosocial challenges. In the broader cancer literature, adaptation is understood as a dynamic process with cancer survivors highlighting coping strategies, social connection, spirituality, and meaning making as key adaptive processes [14], yet these mechanisms remain underexplored in sarcoma research.

This review addresses these gaps by synthesising most up to date qualitative research on the psychosocial impact of sarcoma in adults. Specifically, it focuses on adult experiences, integrates psychosocial impact across emotional, social, relational, and functional domains, and examines adaptation as an ongoing dynamic process rather than a fixed outcome. The review therefore aims to deepen understanding of how individuals experience, make sense of, and respond to the disruptions associated with sarcoma, and the psychosocial needs they identify. The review is guided by the following research questions:

1. How do individuals describe the psychosocial impact of sarcoma?
2. How do individuals describe making sense of and adapting to the psychosocial challenges associated with sarcoma?
3. What psychosocial needs do individuals identify across their experiences of sarcoma?

Through addressing these questions, the review seeks to provide an integrated understanding of the lived psychosocial experience of sarcoma.

## 2 Methods

This systematic review was preregistered with PROSPERO International Register of Systematic Reviews (CRD42024571502, 27/08/2024) and reported in accordance with PRISMA guidelines [15], with adaptations for qualitative synthesis [16] guided by the Enhancing Transparency in Reporting the Synthesis of Qualitative Research (ENTREQ) guidelines [17] (Supporting Information S1).

### 2.1 Design

A qualitative meta‐synthesis was conducted aiming to generate new understandings and identify conceptual gaps, following the principles of thematic synthesis [18]. This involved four stages: (1) systematic searching, (2) study selection, (3) quality appraisal, and (4) thematic synthesis. An inductive analytic stance guided the interpretation and grounded analysis in participant's accounts.

### 2.2 Literature Search

Searches were conducted on 21st of November 2024 across PsycINFO, MEDLINE, CINAHL, and Web of Science, with an update on 30th of December 2025. The search strategy was developed by the lead reviewer with supervision from Senior Lecturers in Clinical Psychology, a Principal Psycho‐oncologist, and librarian support.

Search terms combined two main concepts: (1) Sarcoma and (2) Psychosocial impact (e.g., mental health, wellbeing, adjustment, coping, relationships, stigma, see Supporting Information S2). Backward and forward citation searching of included studies was also conducted.

### 2.3 Study Eligibility Criteria

Studies were included if they: (1) involved adults (≥ 18 years) with confirmed sarcoma; (2) explored psychosocial experiences; (3) used qualitative methods; (4) were peer‐reviewed; and (5) were published in English. To support a focused and coherent synthesis, Gastrointestinal Stromal Tumours (GIST) and Kaposi sarcoma were excluded due to their distinct treatment pathways and psychosocial challenges, including long‐term targeted therapy in GIST and virus‐related stigma and care pathways in Kaposi sarcoma [19, 20]. No limits were placed on publication date. See Supporting Information S3 for full eligibility criteria.

### 2.4 Study Selection and Data Extraction

Records were managed in Rayyan, a systematic review tool, for screening and management [21]. Following duplicate removal, 100% of titles and abstracts were independently screened by two reviewers (RB and ER) against the eligibility criteria. Full‐text articles were 100% assessed independently by the same reviewers. Discrepancies were resolved through discussion with a third reviewer (FOK), with decisions documented.

Data extraction was conducted by one reviewer (RB) for 100% of included studies using a pre‐designed template and independently checked for accuracy by two reviewers (ER and PC), each verifying 50% of the studies. Extracted information included authors, year, country, study aims, design, analytic method, and sample characteristics (e.g., gender age, sarcoma type, treatment status, sarcoma stage). Psychosocial findings were recorded at two levels:

1. First‐order constructs: participant quotations;
2. Second‐order constructs: authors' interpretations and thematic statements

Data were tabulated in Microsoft Excel. Where information was unclear, authors were contacted and given 1 month to respond; if no response was received, studies were retained without the missing data.

### 2.5 Quality Appraisal

Quality assessment was conducted using the Critical Appraisal Skills Programme (CASP) Qualitative Studies Checklist [22]. One reviewer (RB) assessed 100% of studies, with independent assessment by two reviewers (ER and PC), each assessing 50%. The 10‐item CASP tool evaluates methodological rigour and validity across three domains: Section A evaluates study design and methodological integrity; Section B assesses clarity and credibility of findings; and Section C considers the value and applicability of results.

In line with Long et al. [23] recommendation, particular attention was given analytic rigour (‘Was the data analysis sufficiently rigorous’). Following Butler et al. [24] guidelines, each item was rated as Yes (1 point), Can't tell (0.5 points), or No (0 points), producing a total score (maximum 10). Studies were categorised as: High quality (≥ 9), Moderate (7.5–8.5), or Low (< 7.5).

Low‐quality studies were not automatically excluded unless significant ethical or methodological flaws undermined data credibility (a score of < 1 on item 7 [Have ethical issues been taken into consideration?]). Final quality ratings were determined by consensus.

### 2.6 Data Synthesis

A thematic synthesis approach [18], was used to inductively translate diverse qualitative data into descriptive and analytical themes grounded in participant's perspectives. It involved three stages: (1) line‐by‐line coding of first‐ and second‐order data; (2) development of descriptive themes through comparison and grouping of codes; and (3) generation of analytical themes to produces new conceptual insights. Coding and analysis were conducted in NVivo 15 by three reviewers (RB, ER, and PC) with experience in thematic analysis.

### 2.7 Rigour and Reflexivity

Rigour and reflexivity were supported through independent coding, transparent documentation, and iterative team discussions. Reflexive meetings were used throughout the synthesis to examine assumptions, compare interpretations, and enhance analytic consistency. The lead reviewer (RB), a Trainee Clinical Psychologist, also brought prior research experience in sarcoma, which informed familiarity with the clinical and psychosocial complexities of this population. In addition, lived experience of sarcoma within a close family context emerged during the course of this research. This combination of research and personal experience sensitised the analysis of themes of diagnostic uncertainty, mistrust, family impact, disability, and the role of healthcare systems in shaping adaptation. In particular, it heightened sensitivity to how prolonged uncertainty, fragmented care, and visible functional decline may shape meaning‐making for both patients and families across sarcoma trajectory. It also informed attention to adaptation not as a discrete outcome, but as an ongoing process unfolding in the context of cumulative disruption and shifting support needs. These influences were actively reflected upon during analysis through research supervision meetings and reflective journaling, in which assumptions, emotional responses, and emerging interpretations were explicitly discussed and documented to consider how they may have shaped what was foregrounded in the data, particularly themes relating to uncertainty, trust, bodily change, and system‐level support. FOK, an academic Clinical Psychologist with expertise in the intersection of physical and mental health and experience in systematic reviews and thematic analysis, contributed methodological and psychosocial perspectives. LOD, a Principal Specialist Psycho‐oncologist with extensive clinical experience in sarcoma care, contributed specialist insight into the clinical, rehabilitative, and survivorship context of sarcoma. Bringing these perspectives together supported a reflexive analytic process in which interpretations were discussed, challenged, and refined across the team, helping ensure that the conceptual framing remained grounded in participants' accounts rather than in any one professional or personal perspective.

## 3 Results

### 3.1 Study Inclusion

**FIGURE 1:**
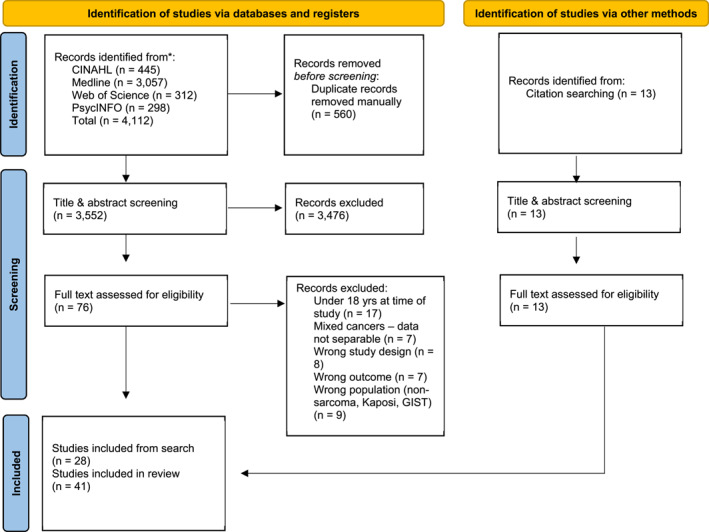
PRISMA flow diagram.

The article selection process can be seen in the PRISMA diagram (Figure 1). A total of 40 studies were included in the final synthesis. Articles were published from 2008–2025. There was a total of 538 sarcoma participants across the included articles (age range 18–80+ yrs, 43% male (*N* = 230)), 48% female (*N* = 258), and 9% not specified (*N* = 50). See Table 1 for study characteristics.

**TABLE 1:** Characteristics of included studies.

ID	Author(s) (year)	Country/study setting	Aim	Findings/themes	Participants	Treatment status	Data collection	Data analysis
1	Almeida et al. (2024) [25]	Portugal/oncology hospital	To enhance the understanding of the QoL challenges of Portuguese individuals with sarcoma.	(1) Interrupted life, (2) learning to live with the sarcoma and (3) need for more individualised care.	*N* = 7; age = 22–78 years; gender = 3(M), 4(F); type = STS (3), BS (4)—(Leiomyosarcoma, Ewing's sarcoma, other sarcoma)	NR	Mixed method—Semi‐structured interview.	Thematic analysis
2	Antalis et al. (2019) [26]	USA/University of Michigan rogel cancer centre	To examine patient perceptions of survivorships care plans (SCPs) and the role of SCPs in overall health management.	(1) Addressing information needs, (2) monitoring for reoccurrence evokes fear (3) SCP as a health management tool	*N* = 8; age = > 18 years; gender = NR; type = NR	NR	Mixed method—open‐ended questions via telephone interview	Thematic analysis
3	Beghean & coffey (2021) [27]	Ireland/irish sarcoma group	To explore biopsychosocial consequences of STS, self‐management strategies, and barriers/facilitators to same.	(1) Consequences of STS and its treatment, (2) perceived facilitators and barriers to self‐management post‐treatment, and (3) self‐management strategies employed	*N* = 7; age = 26–65+ years; gender = 2(M), 5(F); type = STS (myxofibrosarcoma, myxoid liposarcoma, synovial sarcoma, angiosarcoma	Post‐treatment/survivors	Qualitative study—Semi‐structured interview	Thematic analysis
4	Benedict et al. (2016) [28]	USA/metropolitan cancer centre	To explore young adult's discussions of fertility.	(1) Fertility concern, (2) emotions raised when discussing fertility, and (3) managing fertility concerns	*N* = 2; age = 21–24 years; gender = 1(M), 1(F); type = BS (osteosarcoma, ewing sarcoma)	Post‐treatment/survivors	Qualitative study—Semi‐structured interview and focus group	Grounded theory
5	Burgers et al. (2022) [29]	Netherlands/university medical centres & Netherlands cancer institute	To examine the psychosocial challenges in daily life of the growing group of AYAs (adolescents & young adults) with a UPCP (uncertain or poor cancer prognosis).	(1) Feeling inferior to previous self and others, (2) feeling of being alone, (3) sense of grief about life, and (4) loss of control over the future	*N* = 7; age = > 18 years; gender = NR; type = sarcoma unspecified	NR	Qualitative study—Semi‐structured interview.	Grounded theory by corbin and strauss—Constructivist philosophical perspective
6	Burgers et al. (2023) [30]	Netherlands/university medical centres & Netherlands cancer institute	To explore the care experiences of AYAs with a UPCP, with a focus on areas of improvements and to provide oncology HCPs with examples and tools to provide the best possible care.	(1) Trust, (2) tailored communication, and (3) holistic, empathic and open approach.	*N* = 3; age = 28–41 years; gender = 0(M), 3(F); type = STS (leiomyosarcoma, rhabdomyosarcoma), BS (osteosarcoma)	Mixed	Qualitative study—Semi‐structured interview and focus group	Grounded theory
7	D'Alessandro et al. (2025) [31]	USA/quaternary care Children's hospital	To characterise the experiences of individuals living with incurable and indolent STS for more than 2 years.	(1) Managing physical symptoms related to disease/treatment side effects, (2) navigating feelings of guilt and inadequacy, (3) changing illness experience over time, and (4) self‐reflection generating gratitude	*N* = 5; age = 23–45 years; gender = 2(M), 3(F); type = STS (low‐grade fusion‐driven sarcoma; alveolar soft part sarcoma; deficient epithelioid sarcoma; extra‐skeletal myxoid chondrosarcoma; and epithelioid hemangioendothelioma)	Mixed	Mixed method‐ questionnaire with open‐ended questions	Thematic analysis (inductive)
8	Dean et al. (2025) [32]	UK/sarcoma specialist centre—Royal marsden hospital	To explore patient experience of rehabilitation in the surgical pathway for lower limb STS.	(1) Accessing the right services at the right time; (2) ‘communication is key’—Providing knowledge and support to navigate uncertainty; and (3) the importance of person‐centred rehabilitation.	*N* = 8; age = 32–80 years; gender = 4(M), 4(F); type = lower limb STS (well‐differentiated/de‐differentiated lipo‐sarcoma; undifferentiated pleomorphic sarcoma; pleomorphic sarcoma; synovial sarcoma; extraosseous ewing sarcoma; solitary fibrous sarcoma)	Post‐treatment/survivors	Qualitative study—Semi‐structured interview.	Thematic analysis (inductive)
9	den Hollander et al. (2022) [33]	Multiple countries (Germany, Italy, Jordan, Netherlands, UK)	To gain more insight in the HRQoL issues that individuals with uterine STS face.	(1) Physical health, (2) mental health, and (3) social health	*N* = 13; age = 39–61 years; gender = 0(M), 13(F); type = STS (leiomyosarcoma, endometrial stromal sarcoma, rhabdomyosarcoma, sarcoma NOS, other (adenosarcoma, PEComa, undifferentiated spindle cell sarcoma)	Mixed	Mixed method—Semi‐structured interview	Thematic analysis
10	Denissen et al. (2025) [34]	Netherlands/radboud university medical centre	To explain the needs for rehabilitation of patients with BS before and after surgical resection and reconstruction.	(1) Patients have the need to achieve a new normal, (2) patients have the need to be understood, (3) patients have the need to be prepared, (4) patients have a need for optimal conditions for rehabilitation, (5) patients have a need for trustworthy physical therapists, (6) patients have a need for a clear closure from rehabilitation, and (7) patients have a need for access to expertise in the hospital	*N* = 13; age = 19–78 years; gender = 7(M), 6(F); type = BS (osteosarcoma *n* = 6, chondrosarcoma *n* = 5, angiosarcoma *n* = 1, metastasis *n* = 1)	Post‐treatment/survivors	Qualitative study—Semi‐structured interview	Constructivist, interpretative qualitative grounded theory study
11	Dewhurst et al. (2020) [35]	UK/outpatient clinic—Oncology centre	The aim of this research was to explore factors that might influence the physical activity people with an STS undertake as they receive palliative chemotherapy.	(1) Physical activity as an indicator of normality, (2) implications, loss, and uncertainty for the future due to diagnosis and treatment, and (3) the challenge of recognizing support needs as physical activity decline	*N* = 6; age = 53–69 years; gender = 2(M), 4(F); type = STS (6)—(Endometrial sarcoma, inferior vena cava leiomyosarcoma, abdominal rhabdomyosarcoma, retroperitoneal liposarcoma, leiomyosarcoma of the uterus, pleomorphic spindle cell sarcoma adrenal gland)	Active treatment: Palliative chemotherapy	Qualitative study—Semi‐structured interview	Inductive approach based on a construc‐ tionist perspective
12	Eliason et al. (2022) [36]	USA/sarcoma oncology centre, California	To characterize patient experiences of symptoms and impacts of mSS through CE questioning and CD interviewing.	(1) Physical symptoms: Pain, breathing/respiratory, fatigue, and gastrointestinal, and (2) additional symptoms experienced	*N* = 8; age = 21–51 years; gender = 5(M), 3(F); type = STS (metastatic synovial sarcoma—mSS)	Mixed	Qualitative study—Semi‐structured interview was used.	Thematic analysis
13	Fauske et al. (2015a) [37]	Norway/norwegian radium Hospital, Oslo University hospital	To understand the experience of illness through qualitative methodology.	(1) The impracticalities of daily life due to functional impairment, (2) lost opportunities, and (3) identity change.	*N* = 10.0; age = 18–60 years; gender = 7(M), 3(F); type = BS (osteosarcoma *n* = 2, Ewing's sarcoma *n* = 5, chondrosarcoma *n* = 3)	Post‐treatment/survivors	Qualitative study—Semi‐structured interview	Thematic analysis
14	Fauske et al. (2015b) [38]	Norway/norwegian radium Hospital, Oslo University hospital	To understand the long‐ term experiences of osteosarcoma survivors from a sociocultural and psychosocial perspective.	(1) Negative consequences and (2) positive consequences.	*N* = 8; age = 18–50 years; gender = 4(M); 4(F); type = BS (osteosarcoma—Lower extremity)	Post‐treatment/survivors	Qualitative study—Semi‐structured interview	Thematic analysis
15	Fauske et al. (2016) [39]	Norway/norwegian radium Hospital, Oslo University hospital	To explore how visible body changes following surgical treatment affect the life and identity of primary bone sarcoma survivors 3–10 years after diagnosis.	(1) Hiding and (2) exposing bodily deviations	*N* = 18; age = 18–60 years; gender = 11 (M); 7 (F); type = BS (osteosarcoma, Ewing's sarcoma, chondrosarcoma—Hip/pelvis region or lower extremities)	Post‐treatment/survivors	Qualitative study—Semi‐structured interview	Thematic analysis
16	Fauske et al. (2019) [40]	Norway/norwegian radium Hospital, Oslo University hospital	To identify and explore different trajectories that bone sarcoma survivors might navigate during follow‐up.	(1) Back to normal, (2) a new normal, and (3) still struggling	*N* = 18; age = 18–60 years; gender = 11 (M), 7 (F); type = BS (osteosarcoma, Ewing's sarcoma, chondrosarcoma—Hip/pelvis region or lower extremities)	Post‐treatment/survivors	Qualitative study—Semi‐structured interview	Thematic analysis
17	Fauske et al. (2025) [41]	Norway/sarcoma centre—Norwegian radium Hospital Oslo University hospital	To explore how sarcoma survivors experienced communication with and support from healthcare personnel during their outpatient long‐term follow‐up care.	(1) Outpatient follow‐up, (2) psychosocial concerns, (3) information on side effects, and 4) feedback on the checklist.	*N* = 28; age = 20–73 years; gender = 15(M), 13(F); type = STS and BS, not specified.	Mixed	Qualitative study—Semi‐structured interview	Reflexive thematic analysis
18	Gough et al. (2019a) [42]	UK/royal marsden (RM) NHS foundation trust	To explore qualitatively the individual constituents of HRQoL in two groups of patients with advanced STS	(1) Physical domains, (2) psychological domains, and (3) social domains	*N* = 14.0; age = 32–82 years; gender = 5(M), 9(F); type = adavanced STS (leiomyosarcoma, PeComa, plemorphic, sarcoma NoS, synovial, MFH, epithelioid, liposarcoma, fibrosarcoma	Active treatment—Advanced STS	Longitudinal mixed methods—Semi‐structured interview	Thematic & exploratory analysis
19	Gough et al. (2019b) [43]	UK/royal marsden (RM) NHS foundation trust	To explore patient‐centred accounts of the value and timing of prognostic discussions in advanced STS.	(1) Rarity causing prognostic uncertainty, (2) avoiding the negative, and (3) physical symptoms a better prognostic indicator than physician guess.	*N* = 24; age = 19–82 years; gender = 8 (M), 16(F); type = STS (leiomyosarcoma, liposarcoma, epithelioid sarcoma, pleiomorphic sarcoma, fibrosarcoma, MFH, other sarcoma)	Mixed	Qualitative study—Semi‐structured interview	Thematic & exploratory analysis
20	Halkett et al. (2025a) [44]	Australia/community organisation	To explore the potential role a helpline could play in supporting people diagnosed with sarcoma and carers.	(1) ‘The current system is not meeting needs’, (2) ‘holistic support,’ (3) ‘A source of credible information,’ (4) ‘the helpline operator’ and (5) ‘centralised support for my sarcoma’	*N* = 12; age: 21–61 years; gender = 3(M), 9(F); type = STS and BS, not specified	NR	Qualitative study—Semi‐structured interview	Reflexive thematic analysis
21	Halkett et al. (2025b) [45]	Australia/community organisation	To determine the information needs from the perspectives of people with sarcoma, carers and HCPs.	(1) ‘Accessing useful information about diagnosis and treatment’; (2) ‘learning to live with sarcoma’; (3) ‘gaining access to psychosocial support’; (4) ‘connecting with the sarcoma community’; (5) ‘seeking practical support and information’; and (6) ‘obtaining financial support’;	*N* = 18	NR	Qualitative study—Semi‐structured interview	Thematic analysis
22	Hewitt et al. (2019) [46]	UK/hospital in the north west of England.	To gain a deeper understanding of patients' perceptions of treatment for STS, identify concerns throughout treatment, and consider what patients found helpful.	(1) Care process, (2) coping with treatment, and (3) social relations throughout treatment.	*N* = 19; age = 29–84 years; gender = 11(M), 8(F); type = STS (not specified)	Post‐treatment/survivors	Qualitative study—Semi‐structured interview	Thematic analysis
23	Kain et al. (2017) [47]	USA/university of Iowa hospitals and clinics	To determine general categories of information patients want to know at various time points in their care and investigate how patients coped with various physical and psychosocial issues that may occur with sarcoma treatment.	(1) Information about diagnosis and treatment, (2) relationship with the care team, (3) social support, and (4) restoration to ‘normal’.	*N* = 20; age = 22–79 years; gender = 11(M); 9 (F); type = BS (chondrosarcoma and osteosarcoma) and STS (undifferentiated pleomorphic sarcoma, myxofibrosarcoma, leiomyosarcoma, malignant peripheral nerve sheath tumour, fibromyxosarcoma, synovial sarcoma	Post‐treatment/survivors	Qualitative study—Focus group	Scissor‐and‐sort technique/Content analysis
24	Košir et al. (2020) [48]	Canada/sarcoma medical centre, montreal	(1) To develop a better understanding of the affective responses and psychological functioning and identify coping mechanisms.	(1) Changes in mood, (2) worry, and (3) body image concerns.	*N* = 28; age = 24–75 years; gender = 15(M); 13(F); type = STS (lower and upper extremity)	Post‐treatment/survivors	Qualitative study—Focus group & semi‐structured interview	Inductive thematic networks approach and inductive content analysis
25	Lidington et al. (2021) [49]	UK/hospital and local charities	To explore the healthcare experiences of YAs with cancer treated in UK hospitals.	(1) Delay in diagnosis, (2) navigating the healthcare system,(3) health information, (4) variability in fertility preservation discussion and (5) signposting to relevant resources	*N* = 13; age = 25–42 years; gender = NR; type = not specified	Mixed	Qualitative study—Semi‐structured interview and focus group	Thematic analysis
26	Lorimer et al. (2025) [50]	Australia/hospital and local charities	To provide insights into the psychosocial, emotional, and practical challenges that shape the post‐treatment journey for informal carers and adults diagnosed with sarcoma.	(1) It takes a village, (2) I'll Be strong, and (3) navigating life after sarcoma	*N* = 5; age = 21–53 years; gender = 2(M), 3(F); type = STS (epithelioid, retroperitoneal liposarcoma), BS (chondrosarcoma, osteosarcoma)	Post‐treatment	Qualitative study—Semi‐structured interview	Reflexive thematic analysis
27	Magasi et al. (2022) [51]	USA/cancer centres in midwestern United States	To examine how survivors of breast cancer, head and neck cancer, and sarcoma negotiate disability understandings and experiences.	(1) Long‐term effects and (2) disability identity	*N* = 5; age = 22–67 years; gender = 0(M), 5(F); type = not specified	Post‐treatment/survivors	Qualitative study—Semi‐structured interview	Thematic analysis
28	Martin et al. (2023) [52]	UK/hospitals in england and scotland	To describe pre‐diagnostic signs/symptoms and pathways to diagnosis, including where help was sought and what processes were involved prior to the diagnosis.	(1) Experience of the diagnosis process.	*N* = 5; age = 25–65+ years; gender = 5(M), 0(F); type = BS (not specified)	Post‐treatment/survivors	Mixed method—Semi‐structured interview.	Thematic analysis
29	Martins et al. (2019) [5]	UK/hospitals	To explore the experiences of patients with PBC across the UK.	(1) Physical, (2) emotional, (3) social well‐being and (4) role of healthcare professionals.	*N* = 22; age = 25–66+ years; gender = not specified; type = BS (not specified)	Mixed	Qualitative study—Semi‐structured interview and focus group	Framework analysis
30	Martins et al. (2024) [53]	UK/multicenter study in the United Kingdom recruiting through specialist STS clinical teams	To provide an in‐depth exploration of patients' experience of being diagnosed with STS to provide context to the developing outcome measure.	(1) Impact of STS‐ individual and social factors, and (2) Context and processes of care.	*N* = 68; age = 23–82 years; gender = 40(M), 28(F); type = STS (not specified)	Mixed	Qualitative study—Semi‐structured interview and focus group	Framework analysis
31	Parsons et al. (2008) [54]	Canada/mount sinai hospital	To characterise the lived experiences of illness of people with osteosarcoma and to characterize the lived experiences of resuming vocational pursuits.	(1) Illness work, (2) identity work and (3) vocational work	*N* = 14; age = 18–38 years; gender = 8(M); 6(F); type = BS (osteosarcoma—12 lower extremity, 2 upper extremity)	Post‐treatment/survivors	Qualitative study—Semi‐structured interview	Narrative analysis
32	Peairs et al. (2025) [55]	USA/orthopedic oncology clinic	The aim was to explore the lived experiences of patients with sarcoma as pertaining to social determinants of health.	(1) Education access and quality, (2) healthcare access and quality, (3) economic stability, (4) neighbourhood and built environment, and (5) social and community context	*N* = 17; age = 18+; gender = 9(M), 8(F); type = STS and BS, not specified	Mixed	Qualitative study—Semi‐structured interview	Framework method thematic analysis
33	Soomers et al. (2020) [56]	UK & netheralands/Radboud university medical centre (radboudumc), NL, or the royal marsden NHS foundation trust, UK	To investigate the route to diagnosis (RtD) experienced by individuals with sarcoma, including factors contributing to the length of the total interval from the perspective of a patient	(1) Diagnostic interval: Diagnostic phase and effect of centralisation, (2) reflection on diagnostic pathway: Satisfaction with care and impact of delay and (3) recommendations for improvement of the diagnostic pathway	*N* = 15; age = 18–69 years; gender = 6(M), 9(F); type = STS & BS (osteosarcoma, nerve sheath sarcoma, solitary fibrous tumour, leiomyosarcoma uterus, osteosarcoma, solitary fibrous tumour, ewing sarcoma, solitary fibrous tumour groin, leiomyosarcoma, endometrial stromal sarcoma uterus, ewing sarcoma thorax, ewing like sarcoma thorax, liposarcoma, undifferentiated pleomorphic sarcoma, differentiated liposarcoma)	Pre‐treatment and active treatment	Qualitative study—Semi‐structured interview	Content analysis
34	Spears et al. (2008) [57]	UK/Hospital	To explore the emotional support given to individuals with sarcoma by ward‐based nurses.	(1) Use of humour, warmth and positive attitude, (2) inappropriate/unprofessional behaviour, (3) recognition of feelings/needs, (4) continuity and relationship with nurse, (5) practical nurse behaviour and (6) availability and time	*N* = 5; age = 24–37 years; gender = 3(M), 2(F); type = not specified	Not specified	Qualitative study—Semi‐structured interview	Thematic analysis
35	Taylor and Pooley (2018) [58]	Australia/Hollywood functional rehabilitation clinic	To provide insights into sarcoma survivors' thought patterns which impinge on their psyche and their motivational drive to reshape their lives.	(1) Undergoing treatment, (2) surviving sarcoma, and (3) looking forward	*N* = 7; age = 18–44 years; gender = 2(M), 5(F); type = BS & STS (chondrosarcoma, giant cell [a malignant form of giant cell tumour of bone] or Ewing's sarcoma)	Post‐treatment/survivors	Qualitative study—Reflective journaling design	Interpretative phenomenology analysis (IPA) and inductive thematic analysis
36	Taylor and Pooley (2017) [59]	Australia/hollywood functional rehabilitation clinic	To explore sarcoma survivors' thought patterns regarding their post‐operative body image, functionality, and quality of life	(1) Pre‐ and post‐operative body image experiences and mobility concerns and (2) reconstructing a future healthier and happier quality of life	*N* = 7; age = 18–44 years; gender = 2(M), 5(F); type = BS & STS (chondrosarcoma, giant cell [a malignant form of giant cell tumour of bone] or Ewing's sarcoma)	Post‐treatment/survivors	Qualitative study—Reflective journaling design	Interpretative phenomenology analysis (IPA) and inductive thematic analysis
37	van Eck et al. (2021) [49]	Germany, Norway, The Netherlands, Italy, Cyprus, and the United Kingdom	The aim of this study was to assess the unique issues experienced by patients with primary thoracic and primary breast sarcomas.	(1) ‘Physical health’, (2) ‘mental health’, and (3) ‘social health’	*N* = 23; age = 18–79+ years; gender = 8(M), 15(F); type = STS (thoracic sarcoma [liposarcoma, myxofibrosarcoma, rhabdomyosarcoma, sarcoma NOS], breast sarcoma [angiosarcoma, sarcoma NOS]), BS (thoracic sarcoma [chondrosarcoma, Ewing's sarcoma])	Mixed	Qualitative study—Semi‐structured interview	Thematic analysis
38	van Kouswijk et al. (2024) [60]	Netherlands/department of orthopaedics of university medical centre groningen	To identify what factors, influence functional recovery after limb‐salvage surgery (LSS) for lower extremity musculoskeletal tumours (LEMT) from joint patient and HCP perspectives.	(1) Body structures and functions, (2) activities and participation, (3) environmental factors, and (4) expectations	*N* = 11; age = 40–59 years; gender = 2(M), 2(F); type = BS (osteosarcoma, chondrosarcoma), STS (myxoid liposarcoma)	Post‐treatment/survivors	Exploratory qualitative study—Semi‐structured interview with open and closed ended questions.	Deductive and inductive qualitative content analysis
39	Vetchy et al. (2025) [61]	Austria/medical university of Vienna, department of orthopaedics and trauma surgery	To explore how patients experience the time following amputation after primary limb salvage surgery due to musculoskeletal malignancies.	(1) Prosthesis, (2) orthopaedic technician, (3) quality of life, and (4) amputation	*N* = 9; Age = 22–65 years; Gender = 4(M), 5(F); type = sarcoma, unspecified	Post‐treatment/survivors	Qualitative study—Semi‐structured interview	Maryn content analysis
40	Zambrano et al. (2020) [62]	Switzerland/bern university hospital	To identify what motivates survivors of extremital sarcoma to return to work (RTW).	(1) Searching for distraction and wanting to leave the disease behind, (2) the problems with the ‘new’ normal, (3) signs of readiness, and (4) motivating factors and meaning of returning to work	*N* = 15; age = 27–55 years; gender = 7(M), 8(F); type = STS—Extremity, BS—Extremity	Post‐treatment/survivors	Qualitative study—Qualitative survey approach to data collection through an open‐ended questionnaire	Inductive thematic analysis
41^a^	Znajda et al. (1999) [63]	Canada/mount sinai hospital	To identify psychosocial issues experienced by patients treated with surgery and irradiation for STS of the thigh.	(1) Lack of preparedness for the diagnosis, (2) support sought and given, (3) need for more information, (4) changes to daily life, (5) a change in focus from self to ‘I relating to others, ’ and (6) recognition of cancer as a ‘forever disease’.	*N* = 10; age = 40–60 years; gender = 5(M), 5(F); type = STS of the thigh	Post‐treatment/survivors	Qualitative study—An unstructured interview was used.	Unknown—Data analysis involved description of the major categories and subcategories with the intention of finding major themes

Abbreviations: HRQoL = Health Related Quality of Life; MS = mixed sarcoma; NR = not reported; OS = osteosarcoma; QoL = Quality of Life; STS = soft tissue sarcoma; Yas = Young Adults.

^a^ Study 41 was excluded from the final synthesis following quality assessment review.

### 3.2 Quality Appraisal of Included Studies

All included studies were quality appraised (see Table 2) using CASP tool and scored between 7 and 10, with 32 studies being of ‘high quality’, 6 being ‘moderate quality’, and 2 being ‘low quality’. One study Znadja et al. [63] was excluded from the synthesis following quality assessment as it received a score of < 1 on item 7 on the CASP Checklist. Overall, studies demonstrated good congruity between aims, methodology, data collection, and analysis; however, around half provided limited or unclear consideration of researcher‐participant relationships, an important reflexive issue in qualitative research, particularly when examining sensitive psychosocial experiences.

**TABLE 2:** Quality appraisal of included studies.

		Critical appraisal skills programme (CASP) items										
ID	Author (Year)	Q1	Q2	Q3	Q4	Q5	Q6	Q7	Q8	Q9	Q10	Quality
1	Almeida et al. (2024) [25]	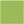	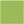	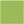	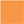	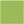	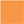	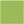	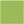	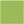	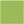	High
2	Antalis et al. (2019) [26]	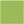	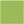	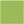	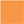	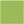	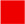	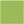	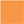	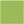	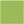	Moderate
3	Beghean & coffey (2021) [27]	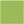	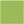	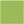	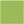	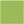	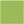	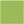	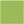	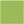	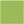	High
4	Benedict et al. (2016) [28]	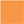	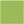	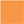	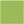	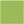	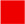	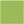	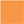	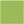	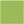	Moderate
5	Burgers et al. (2022) [29]	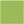	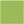	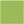	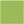	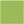	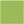	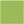	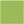	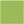	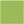	High
6	Burgers et al. (2023) [30]	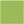	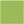	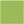	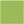	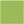	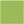	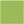	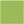	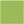	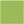	High
7	D'Alessandro et al. (2025) [31]	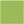	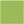	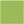	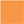	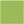	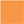	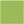	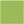	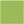	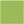	High
8	Dean et al. (2025) [32]	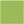	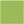	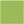	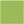	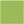	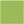	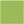	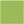	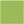	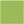	High
9	den Hollander et al. (2022) [33]	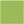	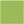	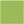	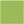	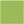	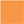	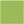	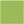	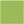	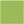	High
10	Denissen et al. (2025) [34]	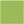	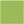	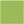	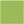	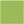	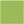	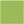	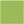	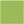	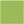	High
11	Dewhurst et al. (2020) [35]	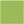	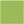	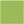	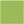	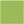	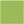	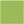	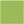	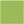	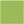	High
12	Eliason et al. (2022) [36]	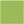	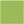	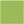	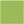	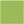	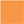	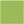	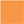	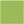	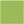	High
13	Fauske et al. (2015a) [37]	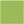	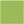	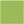	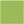	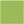	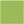	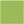	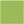	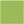	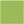	High
14	Fauske et al. (2015b) [38]	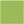	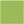	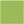	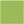	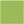	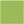	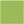	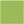	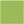	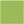	High
15	Fauske et al. (2016) [39]	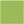	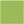	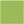	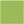	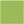	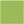	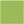	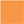	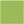	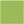	High
16	Fauske et al. (2019) [40]	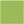	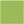	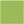	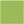	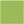	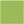	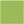	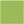	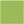	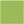	High
17	Fauske et al. (2025) [41]	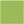	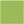	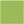	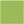	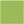	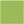	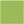	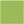	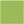	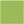	High
18	Gough et al. (2019a) [42]	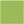	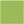	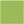	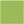	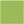	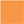	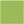	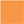	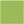	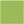	High
19	Gough et al. (2019b) [43]	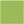	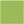	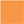	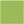	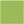	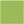	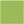	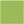	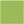	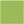	High
20	Halkett et al. (2025a) [44]	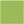	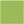	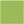	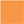	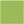	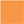	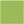	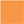	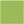	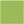	Moderate
21	Halkett et al. (2025b) [45]	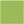	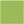	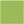	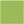	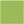	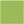	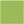	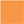	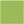	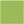	High
22	Hewitt et al. (2019) [46]	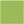	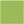	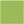	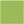	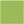	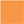	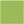	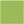	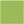	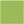	High
23	Kain et al. (2017) [47]	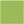	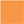	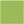	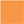	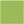	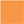	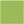	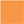	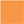	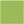	Moderate
24	Košir et al. (2020) [48]	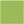	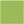	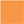	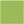	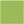	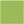	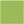	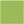	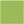	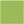	High
25	Lidington et al. (2021) [49]	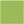	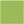	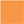	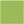	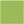	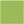	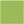	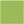	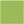	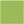	High
26	Lorimer et al. (2025) [50]	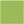	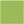	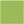	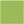	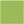	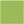	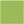	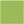	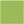	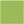	High
27	Magasi et al. (2022) [51]	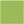	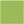	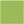	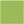	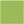	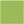	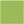	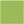	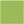	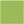	High
28	Martin et al. (2023) [52]	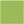	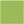	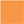	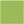	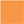	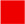	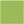	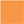	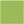	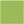	Moderate
29	Martins et al. (2019) [5]	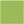	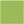	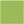	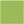	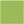	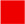	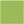	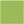	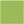	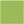	High
30	Martins et al. (2024) [53]	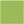	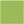	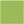	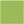	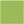	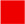	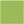	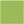	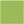	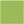	High
31	Parsons et al. (2008) [54]	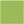	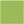	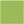	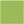	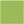	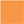	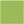	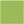	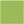	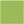	High
32	Peairs et al. (2025) [55]	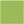	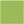	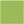	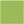	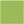	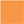	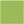	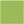	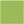	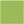	High
33	Soomers et al. (2020) [56]	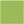	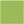	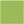	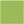	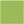	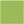	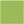	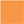	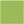	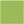	High
34	Spears et al. (2008) [57]	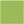	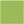	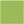	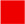	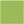	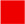	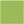	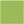	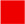	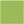	Low
35	Taylor and Pooley (2018) [58]	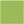	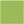	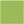	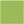	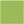	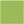	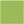	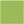	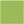	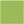	High
36	Taylor and Pooley (2017) [59]	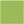	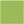	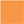	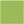	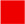	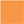	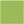	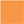	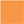	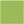	Low
37	van Eck et al. (2021) [49]	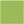	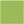	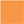	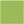	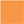	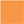	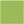	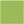	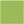	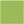	Moderate
38	van Kouswijk et al. (2024) [60]	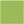	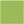	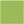	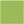	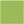	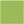	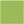	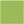	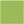	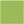	High
39	Vetchy et al. (2025) [61]	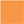	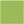	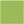	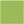	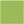	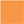	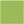	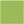	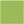	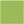	High
40	Zambrano et al. (2020) [62]	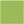	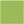	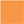	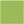	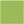	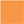	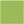	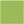	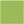	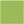	High
41	Znajda et al. (1999) [63]	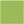	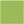	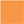	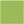	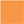	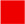	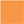	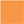	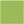	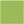	Excluded^a^

*Note:* 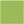 = Yes, 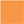 = Can’t Tell, 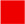 = No. Q1–Q10 correspond to checklist items. Papers were categorized into high‐quality (score 9.0–10), moderate‐quality (7.5–9.0), and low‐quality (7–5.5) studies. Q1. Was there a clear statement of the aims of the research?. Q2. Is a qualitative methodology appropriate?. Q3. Was the research design appropriate to address the aims of the research?. Q4. Was the recruitment strategy appropriate to the aims of the study?. Q5. Was the data collected in a way that addressed the research issue?. Q6. Has the relationship between researcher and participants been adequately considered?. Q7. Have ethical issues been taken into consideration?. Q8. Was the data analysis sufficiently rigorous?. Q9. Is there a clear statement of findings?. Q10. How valuable is the research?.

Abbreviation: CASP = Critical Appraisal Skills Programme qualitative checklist.

^a^ Study 41 has been excluded from the synthesis due to receiving < 1 on item 7.

### 3.3 Review Findings

**FIGURE 2:**
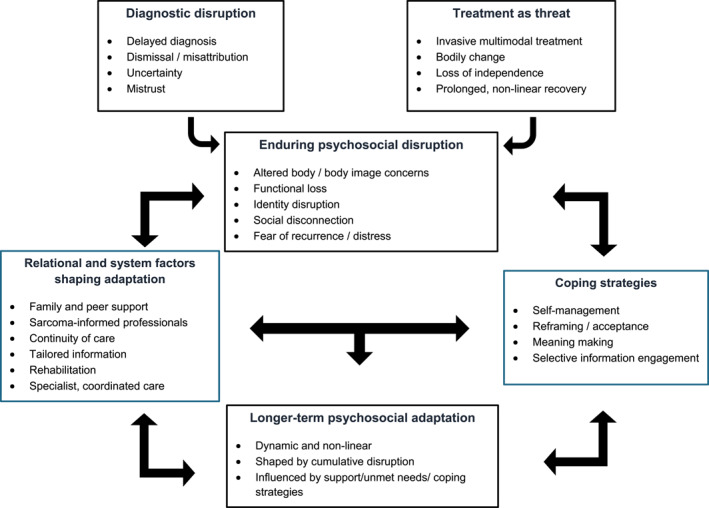
Conceptual model of psychosocial adaptation in adults with sarcoma. Figure 2 illustrates the conceptual model emerging from the synthesis, showing how psychosocial disruption accumulates across the sarcoma trajectory and how long‐term adaptation is shaped by both individual and system‐level factors.

Using the thematic synthesis approach [18] 5 themes and 13 subthemes were identified. All themes were supported by between 14 and 34 studies, including several of high methodological quality (see Table 3). These themes capture the complex psychosocial dimensions of living with sarcoma across the illness trajectory where disruption begins early in the diagnostic pathway, accumulates across treatment and recovery, and extends into longer‐term changes in identity, function, and social life. Across this trajectory, participants also described ongoing efforts to adapt, while being supported or constrained by the resources, relationships, and systems available to them (see Figure 2). A summary of these themes and quotes is presented in Table 4.

**TABLE 3:** Frequency of studies contributing to themes and subthemes.

Theme	Subtheme	Study IDs (referenced)	Frequency (*n*)	Total references (*Σ*)
Theme 1. In the dark—Delays, dismissals, diagnostic dread & mistrust	Fragmented diagnostic journey	**6, 11, 25**, 28, **33, 35**	6	42
	Rare & unprepared—The shock, fear, and uncertainty of a sarcoma diagnosis	**1, 9, 11, 19,** 20**, 24, 32, 33, 35**, 36, 37	11	41
Total of studies contributing to theme 1 (*n*)			14	83
Theme 2. Treatment as threat	The burden of recovery	**1, 3, 5, 7, 8, 9, 10, 13, 15, 16, 17, 18,** 23, **24, 26, 27, 29, 30, 32, 35, 38, 39**	22	115
	The invasiveness of multimodal treatment	**3**, 4, **7, 9, 11, 12, 13, 14, 15, 16, 18, 22, 24, 29, 31, 35,** 36, 37	18	187
Total of studies contributing to theme 2 (*n*)			30	302
Theme 3. No longer the same—Identity loss & altered lives	Living in a changed body—Shame and visible scars	**1, 3,** 4, **9, 13, 14, 15, 16, 24, 27, 29, 30, 31**, 36, 37, **38**	16	88
	The weight of functional loss—Disrupted roles & lost independence	**1, 3**, 4, **5, 7, 9, 11, 12, 13, 14, 15, 16, 18, 21, 24, 27, 29, 30, 31, 32, 35,** 36, 37, **38, 39, 40**	26	301
	Social disconnection—Isolation, stigma & feeling like a burden	**1, 3,** 4, **5, 9, 13, 14, 15, 16, 22**, 23, **24, 26, 27, 29, 30, 31**, 36, 37, **40**	20	118
	The emotional cost of sarcoma—Fear of recurrence & distress	**1,** 2, **3, 5, 7, 9, 11, 12, 13, 14, 16, 18, 22,** 23, **24, 30, 31**, 37, **38**	19	90
Total of studies contributing to theme 3 (*n*)			30	597
Theme 4. What helped along the way‐support & strength in the face of sarcoma	Self‐management—Activity, reframing, & acceptance	**1, 3, 5, 7, 9, 10, 11, 13, 14, 15, 16, 18, 19, 22,** 23, **24, 25, 26, 30, 31, 32, 35,** 36, 37, **38, 39, 40**	27	282
	Relational & system‐level support—Not facing sarcoma alone	**1,** 2, **3, 7, 8, 9, 11, 17,** 20**, 21, 22,** 23, **25, 26, 29, 30**, **32,** 34, **35, 39**	20	197
	Choosing not to know: Preserving hope through selective engagement	**1, 3, 19, 22,** 23, **40**	6	41
Total of studies contributing to theme 4 (*n*)			34	520
Theme 5. What do people with sarcoma need	Diagnostic improvement	**3, 6, 8, 25, 30, 33**	6	33
	Specialist, individualised & integrated sarcoma care across treatment, rehabilitation, and survivorship	**1,** 2, **3, 6, 8, 10, 11, 17,** 20, **21, 22,** 23, **25, 26, 29, 30, 32, 33,** 37, **38, 39**	21	273
Total of studies contributing to theme 5 (*n*)			21	306

*Note:* Frequency (*n*) = number of studies contributing to each subtheme; Total References (*Σ*) = total coded instances per subtheme; Total of Studies Contributing to Theme (*n*) = distinct studies represented within each theme (duplicates removed). Study IDs bolded indicate high quality studies.

**TABLE 4:** Analytical and descriptive themes with representative codes on the psychosocial impact of sarcoma.

Analytical theme	Descriptive theme (subtheme)	Code	Illustrative quote	Article ID/author(s)
In the dark—Delays, dismissals, diagnostic dread & mistrust	Fragmented diagnostic journey	Misdiagnosis	‘It's a hematoma because you've obviously pulled a muscle and you know you've pulled a muscle and it looks much like a hematoma’ they said. […] ‘you don't like to dreadfulize, you don't like to think what ifs. I had pulled muscles in the past and they have been painful, and I have had problems with back muscles in the past through dangerous sports, so I just assumed at the time that they were correct, it was a hematoma.’	33 Soomers et al. 2020 [56]
		Long diagnostic process	‘…[They took] a biopsy 3 times. They punctured 3 times, so they would have enough, but they hadn't because it turned out they were still in doubt between a chondrosarcoma and an osteosarcoma.’	33 Soomers et al. 2020 [56]
		A waiting game	‘… it took lots of tests to determine I'd a giant cell tumour… but that was just the start of the waiting… there was lots more waiting for them to determine what to do and how to make it better… it felt like it took forever.’	35 Taylor and Pooley 2018 [58]
		Rarity of sarcoma	‘When you dig into the evidence, obviously sarcoma is a rare tumour, but even so, there's a few studies. As a patient, you're always trying to figure out which one of those study groups reflects you, because they don't.’	32 Peairs et al. 2025 [55]
	Rare and unprepared: Shock, fear & uncertainty	Shock and emotional distress	‘As a surfer and a soccer player for the whole of my life… the biggest initial problem I had when I found out I had a Ewing's sarcoma in my lower right leg… was the shock… of being told that I'd likely lose my right leg at the knee…’	36 Taylor and Pooley 2017 [59]
		Pervasive sense of uncertainty	‘And I think I still don't quite understand what the difference between sarcoma and so‐called normal cancer is. Also, specifically to sarcoma, because the other thing that you hear a lot from the doctors, it's not your typical cancer, so what is typical cancer and how…Because the population is so small, it's just understanding how big are the risks if you do this or you don't do that? Because there's just not a lot of data, there's not a lot of knowledge, it's hard to understand myself, but it's also hard to almost talk about it, because you're not quite sure.’	9 den Hollander et al. 2022 [33]
		Fear	‘It was difficult from the moment of the result at the hospital until the scans. Especially after the scans I was wondering: “Is it somewhere else, how bad is it?” I thought I was dying.’	33 Soomers et al. 2020 [56]
Treatment as threat	The invasiveness of multimodal treatment	Fatigue	‘You just have no energy, it's not that you want to sleep particularly it's just you feel as though your legs won't get you there….’	11 Dewhurst et al. 2020 [35]
		Fertility	‘That made me feel really isolated from people (…) I've always wanted a family, so that has been probably the biggest thing that I've had to overcome’	29 Martins et al. 2019 [5]
		Shortness of breath	“That is simply just walking uphill or if I'm carrying heavy shopping bags then I will get a little bit out of breath, which I didn't used to (pt 12, 54 years, leiomyosarcoma)”	9 den Hollander et al. 2022 [33]
		Surgical pain	‘“The doctor removed the bottom floating ribs and a mat has been placed in it”. When I turn around, I can feel it stinging…’	37 van Eck et al. 2021 [49]
		Fear of limb loss	‘I kept touching my leg to make sure that it had not been amputated. (Participant 1; female, 70 years, minor surgery)’	24 Košir et al. 2020 [48]
	The burden of recovery	Recovery expectations and challenges	‘My expectations were, well, “you get the prosthesis, you learn to walk again by attending physical therapy, and then it will be all right”. But that is not really how it goes; it is just different’	38 van Kouswijk et al. 2024 [60]
		Loss of independence	‘I wished the sarcoma had never happened to me… the sarcoma had eaten away my bone and the bone broke. I had to move back in with my mum and dad as I couldn't pick my daughter up let alone dress, feed, or care for her… I couldn't put any weight on my leg. My parents had to do everything and this was a huge loss of my independence.’	36 Taylor and Pooley 2017 [59]
		Impairment on day‐to‐day activities	‘These last medications have made me fat, I lost many muscles in the legs, and also a little bit in the arms, but not so much. But basically, I lost my autonomy and mobility. I have more and more difficulty with these normal things in life, like taking a shower.’	1 Almeida et al. 2024 [25]
		Ongoing complications	‘To get physically back and I don't know that I realized the complications of some of the nerve damage, and scar tissue damage and things like that I have had to do extra kind of alternative massage therapy’	23 Kain et al. 2017 [47]
No longer the same—Altered lives & loss of self	Living in a changed body—Shame & visible scars	Negative view of body	‘I feel, in all reality, very ugly when I am naked. I had girlfriends who say that they like me regardless, but I don't really believe them because they might just say that because they don't want me to be hurt or something like that. […] I am more daring now but still afraid. Afraid that they will reject me because of my ugly scars.’	16 Fauske et al. 2016 [39]
		Hiding self	‘When people stared out of curiosity that's when I became very self‐conscious of the way I walked. The fact is that back then I limped and walked like an older person and that was embarrassing to me. I felt less confident because my leg looked “funny”… and, random strangers would ask me rude questions about my limp. They'd want to know all of the gory details… so that's when I started to create an appearance of being normal. I wore jeans 365 days a year and tried not draw any attention to myself. I never wore shorts or short skirts’	36 Taylor and Pooley 2017 [59]
		Others' perceptions of their bodies	‘The thing I noticed was how the adults stared at me. The children approached me and asked me what happened, and I said that I had an operation. That was totally fine, and they did not stare at all. How the adults were staring at me, on the other hand, was something that really caught my attention.’	16 Fauske et al. 2016 [39]
		Shame of looking different	‘I just want to look normal. I don't want to look like I have one breast. I don't want to look disabled; I want to be normal. . . . Mentally I was probably at the worst point of my time because I couldn't stop crying…’	27 Magasi et al. 2022 [51]
	The weight of functional loss—Disrupted roles & disability	Functional impairment	‘I have no control. If I go out without crutches, I am afraid of falling. The risk of falling is much greater if I were to lose my foothold with the healthy leg. Then I have no chance of standing up on my feet with just the injured leg.’	13 Fauske et al. 2015a [37]
		Mobility	‘I have less muscle power on the left side because I have fewer muscles. I do exercise regularly, but so many muscles were removed at the time, there is simply no room for improvement.’	38 van Kouswijk et al. 2024 [60]
		Physical disability	‘My functional impairment is, in fact, more than what people can see from watching me walk…I cannot get anything done (on crutches). […] I cannot carry anything; I cannot take anything with me. I have to put everything in a rucksack.’	14 Fauske et al. 2015b [38]
		Changing roles and relationships	‘Um I'm having to accept help with the housework because it's I just you know I mean I'm keeping the basics done um and that would be fine but you know I've got quite a big family…I just can't do all the things I would normally do.’	11 Dewhurst et al. 2020 [35]
		Losing their identity	‘I felt very down…I can't always get down on the floor and play with my children…I sometimes feel like I'm a bad mom.’	7 D'Alessandro et al. 2025 [31]
		Work disruptions	‘…I went back to work probably about a year after. [I] asked them if I could have my job back. The human resources guy said, “yeah, no problem, sure, says to hire you back.” so I waited. He's like, “at the end of the week we'll call you.” that week passed. Another week passed…But basically, they weren't going to give me my old job…I was trying to get a job everywhere. I had resumes everywhere…’	31 Parsons et al. 2008 [54]
	Social disconnection—Isolation, stigma & feeling like a burden	Experience of loneliness	‘It's very lonely because even when you've got all your family around at the end of the day it just comes down to you, doesn't it?’	22 Hewitt et al. 2019 [46]
		Feeling like a burden	‘I can't always get everything off my chest, because I don't want to burden my children with it every time.’	37 van Eck et al. 2021 [49]
		Social life	‘No, people forget a little. Planning things that I cannot attend, and those things. [….] I think that it is obvious that people do so because they do not think that I am disabled and cannot manage what they do. And then, I become sad.’	14 Fauske et al. 2015b [38]
		Not feeling understood	‘I don't want to play like I'm being the victim, but … it was just some people just not even acknowledging it [the impact of sarcoma] at all’	26 Lorimer et al. 2025 [50]
	The emotional cost of sarcoma: Fear of recurrence & distress	Fear of recurrence	‘I never felt cured either, it is known that it is a tumour that can come back quickly.’	37 van Eck et al. 2021 [49]
		Depression	‘I always tried to push myself to do better, to get out of my mood swings, my negative moments and so on. […] after the surgery, it's like my life has collapsed. I Thought I could not do anything with my right hand. […] I was a little depressed.’	24 Košir et al. 2020 [48]
		Worry & fear of death	‘If I go to bed on time, at 10 p.m., I don't sleep and I am afraid of death. So I sleep badly. I go to bed when I feel tired.’	9 den Hollander et al. 2022 [33]
		Lasting negative impact	‘I would come here to the hospital to do my treatments, and always ask myself why …. There were so many people without legs …nothing convinced me, only time helped, and it took 2 years.’	1 Almeida et al. 2024 [25]
What helped along the way—Support & strength in the face of sarcoma	Self‐management: Activity, reframing & acceptance	Preserving a sense of normality	‘when somebody tells you have cancer, normality flies out of the window, I want to keep things as normal as possible for them, you know, my youngest son is in the last year of his degree…, um my other son has um emotional mental anxiety difficulties…so it's important to try and keep as close to normality as possible.’	11 Dewhurst et al. 2020 [35]
		Increasing exercise	‘The gym instructor said to me that my leg's okay and that I just needed to trust my leg because it would support me. She told me what I shouldn't do though was to not rely on my crutch…I asked her if I would need to do my exercises every day for the rest of my life and she replied: Yes… so now I've done more exercises in these last few weeks than I've done over the past 17 years after having my tumour on my left leg…’	36 Taylor and Pooley 2017 [59]
		Accepting the new normal	‘You want to be back to normal as fast as possible and it's not that way. I Think you know you gotta find your new normal. You know it's not, you're not going to be like you were before but you're going to be a new normal. So you find a good goal where you can cope and you go on with life.’	23 Kain et al. 2017 [47]
		Active coping versus rumination	‘… I think the “feel good factor” in physically feeling that you have the upper hand is probably as important as an awful lot of other things you are doing… if you are giving all your body the good endorphins and that sort of thing, rather than looking at a (medical) prognosis and saying “It's not good “and” I wonder how long I'm going to last” you know…’	19 Gough et al. 2019b [43]
		Accepting the body	‘I've become less aware of the difference and most days I don't think about it at all. I've got used to being slow and wonky… and I don't have any issues with the look of my leg. I'm used to it!.’	36 Taylor et al. 2017 [59]
		Reframing experiences	‘Cancer has shown me how far past my limits I can push myself…Real pain, real love, real discipline, real courage. Not just in me, but in others…’	7 D'Alessandro et al. 2025 [31]
	Relational & system‐level support—Not facing sarcoma alone	Family support	‘My wife is my rock. Sometimes she's also the person that says “come on, let's not sit around and mope around. Let's try and get going”…’	8 Dean et al. 2025 [32]
		Social support	’I think because they (patient support groups), they sort of understood how I was feeling, whereas nobody else really did, well they can't can they? My family members didn't really understand what was going on in my head’	22 Hewitt et al. 2019 [46]
		Positive relationships with health care professionals (HCPs)	‘I was about to start bursting into tears and (patient makes crying noise) and so this lovely nurse came walking by and she says to me, well she looked at me and that was it…we chatted and it just made me feel better…’	34 Spears et al. 2008 [57]
	Choosing not to know: Preserving hope through selective engagement	Avoiding prognosis discussions	‘…They never mentioned it, and I've never asked. I don't want to know. I have a treatment plan and I will stick to it. I need to be positive and an ‘expiry date’ doesn't give me that…’	19 Gough et al. 2019b [43]
		Shifting focus away from negativity	‘Putting it out of my mind completely … there's no point in sitting here and biting your nails and thinking.’	3 Beghean and Coffey 2021 [27]
		Choosing not to know prognosis information	‘I don't recall hearing that and I don't think I would have wanted to know [prognosis]. I just wanted to know what I was up against, what the plan was and… how to get through it.’	23 Kain et al. 2017 [47]
What do people with sarcoma need?	Diagnostic improvement	Lack of awareness of sarcoma	‘Possibly my doctor himself should have pushed slightly more rather than saying: Yes, that's fine, you can leave it until after your holidays, [the GP] should've possibly said: “No, let's fix it beforehand”…’	27 Soomers et al. 2020 [56]
		Diagnostic delays	‘…Multiple instances of waiting for doctor, oncologist, and hospital waiting rooms (e.g., waiting for the results…waiting for a referral to an oncologist; waiting for appointment dates to complete the additional tests…waiting for a return appointment with the oncologist to find out the tests results and informed of the likely treatment options’	29 Taylor & pooley, 2018 [58]
		Need for more information about sarcoma	‘There should be more information regarding the disease and clear orientations like you will do this, you will do that. Sometimes there is little information, and you must wait 2 months for the appointment.’	1 Almeida et al. 2024 [25]
		Fighting for diagnosis	‘It makes you feel like they're just as invested in your health and your recovery as you are. So, it makes you feel better, which then makes it feel more important to help prove them right.’	25 Lidington et al. 2021 [49]
	Specialist, individualised & integrated care across treatment and survivorship	Clear information and guidance	‘It actually is important at the start of the trajectory that you actually have a conversation with someone speaking about like, how will the coming treatment trajectory be and what can you expect in the medical area? What can you expect in the rehabilitation area?’	10 Denissen et al. 2024 [64]
		Consistent access to information and care	‘Me and my parents have a certain difficulty in having the information about my case. There are many professionals in the team and it's a little tricky to have the information about my situation … to have all the information. One thing my mom suggested was to have one person who should be responsible for communication with the patient, always the same person who gives you all the information.’	1 Almeida et al. 2024 [25]
		Trust in HCPs	‘… I was halfway through the trajectory [of rehabilitation] before you actually find out. If you go to the physical therapist for the first time, you cannot judge, because he needs to get a chance. But afterwards I thought, they are not specialized in this, they don't completely know.’	35 Taylor and Pooley 2018 [58]
		Inadequate access to specialised rehabilitation	‘The primary care physical therapist is too inexperienced. Sounds tough, but it is true. Don't get me wrong, I was very glad about him, he helped me back on my feet. (.) but I missed training stability there. I'm missing quite a few muscles, so other muscles had to take over. (…) I didn't train that enough back then. A rehabilitation centre is better qualified for that.’	37 van Kouswijk et al. 2024 [60]
		Lack of follow‐up	‘Even post getting home, again no psychology recommendation given or asked: “Would you like [access to services]?”…I went and hunted for all that stuff myself and I just thought how so little follow‐up there has been. It's all about while you're in there and it's the afterwards your kind of left.’	20 Halkett et al. 2025 [45]
		Time for conversations	‘There should be someone with more time for conversation or more prepared to understand what's going on’	1 Almeida et al. 2024 [25]

### 3.4 Theme 1: In the Dark—Delays, Dismissals, Diagnostic Dread and Mistrust

Across studies, participants described the diagnostic process as prolonged, confusing, and saturated with uncertainty. Two subthemes captured these experiences:

#### 3.4.1 Fragmented Diagnostic Journey

The pathway to diagnosis was often characterised by misattributed symptoms, repeated consultations, and delayed referrals.

I kept going to the doctors and he just kept giving me painkillers…I think my GP let me down a bit… having to fight with him to get referrals… That was probably the hardest bit.

(Study 28) [52]

#### 3.4.2 Rare and Unprepared: Shock, Fear, and Uncertainty

Receiving a sarcoma diagnosis was often abrupt and devastating, intensified by limited prognostic clarity and low public awareness.

…I was just so surprised after everything being… “yes, yes everything looks good, everything looks good”. Then bang….

(Study 33) [56]

Participants described struggling to process unexpected news and to communicate it to others who had never heard of sarcoma. These early experiences of uncertainty, shock, and limited understanding appeared to shape how participants entered treatment, often carrying forward a sense of vulnerability and loss of control.

… it’s hard to understand myself, but it’s also hard to almost talk about it, because you’re not quite sure.

(Study 9) [33]

### 3.5 Theme 2: Treatment as Threat

Building on the uncertainty and disruption described in diagnostic phase, treatment was widely described as invasive and overwhelming, both physically and emotionally. Three subthemes capture these experiences:

#### 3.5.1 The Invasiveness of Multimodal Treatment

Participants across studies described chemotherapy, radiotherapy, and surgery as highly invasive experiences that reshaped their bodies and lives. Fatigue was the most frequently reported side effect, often described as debilitating.

It wears me out. It wears me out.

(Study 12) [36]

Surgical procedures, often involving limb‐sparing surgeries, extensive resections or at times amputations, were described as permanently life‐altering. For many, the fear of potential limb loss or loss of function was particularly distressing.

I think that just the fact that I couldn’t be physically active…it would be the beginning of the end sort of thing ….

(Study 11) [35]

#### 3.5.2 The Burden of Recovery

Recovery from treatment, specifically surgery was described as arduous, prolonged and multifaceted process extending beyond physical healing.

I went from being independent to being dependent… I had to have help getting into the shower and even dressing myself… I just felt like crying or screaming!.

(Study 36) [59]

Persistent complications such as pain and slow wound healing prolonged recovery and challenged expectations of returning to normality:

It was an enormous recovery physically; afterwards it took me I would say a good year to physically recover….

(Study 26) [50]

Others experienced recovery as a challenging period, where anticipated relief often gave way to more uncertainty and the realisation that a return to ‘normal’ was no longer possible.

The only thing worse than going through cancer treatment is after cancer treatment… that first month or two after treatment was like oh my gosh, you’re relearning your whole life….

(Study 27) [51]

Right, that’s it, you’re cured. Let’s get back to normal now,’ and I thought, ‘No, I’m not cured’.

(Study 30) [53]

For many, these recovery experiences marked not an end point, but a transition into longer‐term bodily, functional, and psychosocial changes that shaped life beyond treatment.

### 3.6 Theme 3: No Longer the Same—Altered Lives and The Loss of Self

Extending the disruption described in earlier themes, this theme, the most frequently referenced across studies, reflects the profound psychosocial transformation experienced by many individuals with sarcoma. Participants described feeling ‘no longer the same’, as sarcoma and its treatment disrupted their bodies, identities, and social roles. Four subthemes capture these experiences:

#### 3.6.1 Living in a Changed Body—Shame and Visible Scars

Alterations to the body, such as deep surgical scars and amputations, were described as profoundly disruptive to body image and identity with many trying to conceal marks or avoid public spaces.

… I don't wear shirts anymore with short sleeves…I guess I'm a little bit…embarrassed about it.

(Study 24) [48]

#### 3.6.2 The Weight of Functional Loss—Disrupted Roles, Lost Independence, and Disability

Participants described the profound impact of functional loss on daily life, independence, and identity.

For example, making dinner on crutches and trying to carry hot pans is absolutely hopeless.

(Study 13) [37]

Physical limitations disrupted participants' roles as parents, partners, employees, and athletes with many now identifying as *‘being disabled’,* unable to fulfil former roles or sustain the same relationships.

I’m embarrassed because I could do this before… I feel a little less cool, less sporty and somewhat less of a man. […].

(Study 13) [37]

My daughter has a disabled mother, and she is often sad when I cannot be with them.

(Study 14) [38]

#### 3.6.3 Social Disconnection—Isolation, Stigma, and Feeling Like a Burden

Many participants described a deep sense of social disconnection, shaped by both perceived visible bodily changes and practical constraints of functional loss.

Before I had cancer, I had a lot more friends. After I had to quit football, I lost contact with many….

(Study 14) [38]

No, people forget a little. Planning things that I cannot attend…they do not think that I am disabled and cannot manage what they do.

(Study 14) [38]

Participants described being viewed through their impairments and struggling with other's inability to grasp the lasting effects of sarcoma.

It has taken me two years to admit it… I’m disabled. I’m disabled because of people staring at me….

(Study 27) [51]

Many described how concerns about the emotional impact of sarcoma on loved ones contributed to feelings of guilt and distress, shaping how they experienced their relationships.

They [family] start crying and ask why? Nobody knows why, so I felt guilty for hurting them [after sharing diagnosis].

(Study 37) [49]

#### 3.6.4 The Emotional Cost of Sarcoma: Fear of Recurrence and Distress

Participants described the enduring emotional weight of living with sarcoma, marked by persistent fear, anxiety, and sadness that extended well beyond treatment. Fear of recurrence was the most pervasive and enduring emotional impact across studies.

Any lumps, bumps, that kind of thing, you start to think, “Oh no, what if that’s another recurrence?”…You know, every time you go for a scan, every time you go for a follow‐up appointment….

(Study 30) [53]

For many, this fear was compounded by the rarity of sarcoma, unpredictable course, and the absence of clear prognostic markers.

With the breast cancer, I still had the false safety of well, it might go wrong, but it will take a while. I still have some time. And with this one…it really feels like a race against time….

(Study 37) [49]

Alongside these fears and anxiety, many participants described profound sadness, and hopelessness in the aftermath of treatment.

From the moment I had operated on my leg, I became a different person, and it affected me a lot …, I feel sad, I am no longer the person, that I was….

(Study 1) [25]

### 3.7 Theme 4: What Helped along the Way—Support and Strength in the Face of Sarcoma

Among the ongoing uncertainty, altered identity, social, emotional and functional disruption, participants also described a range of ways of coping, responding, and adapting over time.

#### 3.7.1 Self‐Management: Activity, Reframing and Acceptance

Participants described self‐management strategies as vital to preserving a sense of normality. Exercise, diet, clothing choices, and planned daily activities were used to actively regain strength and normality.

I want to be as normal as I can be. I’m wearing long trousers in the summertime to cover the scar […] By doing this, I am first and foremost (participant’s name) and not a former cancer patient.

(Study 15) [39]

Alongside these practical efforts, participants described consciously reframing their experiences as a form of meaning‐making—finding acceptance, gratitude, changed perspectives, and in some cases faith, as sources of strength.

…After having had cancer, I have become more positive…. What I went through, and the fact that I was as strong as I was, gave me more zest for life….

(Study 13) [37]

Other participants described actively seeking information about sarcoma and its treatment as a way to prepare, regain a sense of control, and reduce uncertainty.

I said how long have I got…I felt desperate to know…it was very hard to make any decisions or plans, unless you really know….

(Study 19) [65]

#### 3.7.2 Choosing Not to Know: Preserving Hope Through Selective Engagement

In contrast to those who sought detailed information to prepare and feel in control, other participants described deliberately limiting engagement with illness‐related information or discussion as a way to preserve hope and prevent sarcoma from dominating daily life.

… if someone asked me anything [reference to sarcoma], I would say immediately … You can talk about whatever you want but not about that…Whoever it is. I don’t like to talk….

(Study 1) [25]

#### 3.7.3 Relational and System‐Level Support—Not Facing Sarcoma Alone

Participants emphasised the importance of both personal and professional support in coping with sarcoma and navigating its uncertainty.

Family is a big deal too…I came down for radiation everyday five days a week and I had a different driver every day, which was remarkable.

(Study 23) [47]

Beyond family, support from peers with sarcoma was highly valued, providing a level of understanding not found in family or general cancer groups.

When you go to general cancer groups, people have got so many different experiences, whereas we’re all sarcoma. Yes, we’ve all got, maybe, different types of sarcoma… but it’s still sarcoma…Nobody’s heard about it before they have it and we’ve got that in common. I find that just hugely beneficial.

(Study 30) [53]

Supportive relationships with healthcare professions were also central in providing reassurance, information, and continuity of care. Adaptation was described not only as an individual process, but also as something shaped by support from others and by interactions with healthcare systems.

It makes you feel like they’re just as invested in your health and your recovery as you are. So, it makes you feel better…[when talking about rehabilitation].

(Study 8) [32]

### 3.8 Theme 5: What do People With Sarcoma Need?

Participants also described unmet needs across the trajectory, highlighting how adaptation could be hindered when timely diagnosis, clear information, and specialist, individualised care were not available across treatment, rehabilitation, and survivorship.

#### 3.8.1 Diagnostic Improvement

Participants highlighted the need for faster, more reliable diagnostic pathways and greater awareness of sarcoma among both the public and healthcare professionals.

Everything could have gone faster if people had been more aware that this could be sarcoma.

(Study 33) [56]

the poor sister of cancers [sarcoma], like Cinderella in the fairytale, being the little one who got nothing.

(Study 3) [27]

#### 3.8.2 Specialist, Individualised and Integrated Sarcoma Care Across Treatment, Rehabilitation, and Survivorship

Participants strongly emphasised the need for specialist, coordinated, and individualised care throughout the sarcoma trajectory. Having confidence in a knowledgeable and experienced team was described as central to feeling safe during treatment.

I had every faith in [surgeon’s name], he’s a sarcoma specialist. So, I actually had, immediately, confidence in, you know, the team that were going to be looking after me.

(Study 30) [53]

In contrast, those who received care in non‐specialist settings described feeling abandoned and uncertain about who was responsible for their treatment and recovery.

I had been back to [residential location], no CNS [clinical nurse specialist], no nothing. GP said she’d never met a sarcoma patient before. People didn’t know what it was, so I felt like an orphan really….

(Study 30) [53]

People don’t know about sarcoma. Hospitals don’t, wherever I go…it’s not common and no one knows about sarcoma.

(Study 8) [32]

Clear, consistent, and tailored information across all stages was repeatedly identified as a key need, particularly post‐treatment, when many participants felt ill‐prepared for recovery and rehabilitation.

But for me, … we spoke quite regularly about the surgery, but not after… And if you know what the impact is afterwards, then I would have wanted to know more, like…My knee will be taken out, I will get a new knee and what will happen then?.

(Study 10) [64]

Equally important was access to specialist and individualised rehabilitation care.

For me, if the [care in the] rehabilitation center is how I experienced it, it is better to go home. It did not have any added value. (…).

(Study 10) [64]

…she [healthcare professional] compared my amputation to some nerve damage she had in her hand…it was clear to me that she hadn’t dealt with a sarcoma patient.

(Study 20) [45]

These accounts suggest that psychosocial adaptation in sarcoma was shaped not only by the disruptions individuals faced, but also by the extent to which care systems were able to respond to their ongoing informational, rehabilitative, and supportive needs.

## 4 Discussion

This systematic review synthesised qualitative evidence on the psychosocial impact of sarcoma in adults, drawing on 40 qualitative studies of predominantly high methodological quality. Across studies, consistent findings strengthen confidence in the generalisability of the core themes at a conceptual level. The themes identified show how sarcoma is experienced as a cumulative and ongoing psychosocial disruption. This disruption often began early with prolonged and fragmented diagnostic pathways, intensified through invasive treatment and difficult recovery, and extended into long‐term changes in identity, embodiment, functioning, and social life. At the same time, individuals described actively adapting through self‐management, meaning‐making, selective information engagement, and support from others. This synthesis highlights adaptation as a dynamic process unfolding alongside persistent disruption and unmet needs across the sarcoma trajectory [66, 67].

Across the synthesis, psychosocial impact unfolded along a trajectory that often began ‘in the dark’ (Theme 1). Diagnostic pathways were prolonged and fragmented, involving misattributed symptoms and delayed referrals, generating uncertainty and mistrust consistent with findings across other rare cancers [66]. Diagnostic delay functioned not only as a temporal challenge but as an early psychosocial rupture, shaping fear and complicating individuals' efforts to make sense of sarcoma from the outset [33]. These early disruptions appeared to set the tone for subsequent experiences, shaping how participants encountered treatment and later made sense of ongoing psychosocial change.

Treatment was experienced not as a resolution but as an ongoing threat (Theme 2), with multimodal treatment described as invasive physically and emotionally. Surgery, chemotherapy, and radiotherapy resulted in permanent bodily change and reduced independence, with pain, fatigue, scarring, and limb loss becoming enduring reminders of survival at a significant personal cost [5, 65]. Recovery was rarely linear and instead represented continued vulnerability rather than closure [68]. The findings further suggest that delayed or disrupted rehabilitation may hinder adaptation, particularly where oncological priorities conflict with functional restoration. These tensions may be especially salient in sarcoma given the diversity of treatment pathways and the prolonged demands of multimodal care [69]. In this way, treatment and recovery did not mark a transition out of disruption, but often deepened it, extending uncertainty and vulnerability into life beyond active care.

Post‐treatment life was marked by enduring physical, psychological, and social change with many describing a sense of being ‘no longer the same’ (Theme 3). A central tension in this synthesis was a paradox of visibility and invisibility: although sarcoma is rare and poorly understood, its consequences were often highly visible through scars, prostheses, mobility aids, and functional limitations. Body image issues were therefore pronounced, contributing to shame and diminished confidence [37, 51]. Functional impairment further disrupted identity by threatening independence and valued roles, including parenting, relationships, social activities and careers [25, 59]. Inability to engage in childcare, household tasks, work, or previously valued physical activities appeared to intensify loss and was consistent with patterns associated with poorer psychosocial adjustment and quality of life [8, 70].

These disruptions were further compounded by social isolation, stigma, and persistent emotional distress. Across studies, a profound sense of ‘social disconnection’ became evident with participants frequently withdrawing from social situations not only due to physical limitations, but also because they felt misunderstood or reduced to their impairment. Emotional distress, often described as an ‘emotional cost of sarcoma’, encompassed fear of recurrence (FoR), anxiety, and low mood with FoR emerging as particularly salient and intrusive, echoing wider evidence identifying it as a major concern for people living with sarcoma [71]. This synthesis demonstrates how distress is sustained through the intersection of lasting physical change, social misrecognition and stigma, and the ongoing effort to manage how sarcoma is seen by others. As such, adaptation appeared to involve ongoing efforts to manage not only functional and emotional consequences, but also the social meaning of living in a changed body. Comparative evidence suggests that these interlinked challenges particularly those related to rarity, disability, and visible bodily change following intensive treatment, may be especially salient in sarcoma [7, 66].

Despite these profound disruptions, individuals identified ‘what helped along the way’ (Theme 4). Adaptation emerged as a response to cumulative disruption and was described as a dynamic, non‐linear process involving active self‐management (e.g., physical activity, lifestyle adjustment) alongside cognitive strategies such as reframing, acceptance, and meaning making. These approaches appeared to support agency amid ongoing uncertainty, with distress and adaptation often co‐existing. For some meaning‐making involved recognising personal strength or changed priorities, aligning with broader evidence on post‐traumatic growth (PTG) in cancer populations [72, 73].

While there is currently no PTG research in sarcoma, previous sarcoma reviews suggest that psychosocial wellbeing can co‐exist with substantial functional disruption, supporting the view that positive change may occur alongside ongoing impairment rather than following its resolution [6]. Adaptive variability was also reflected in patterns of information engagement, ranging from active information‐seeking to deliberately ‘choosing not to know’, consistent with evidence that selective engagement can be adaptive when aligned with coping preferences [74]. Adaptation was further shaped by ‘relational and system‐level support’. Family support, peer connection, and continuity with knowledgeable and compassionate healthcare professionals appeared to facilitate coping and meaning‐making, while difficulties navigating fragmented services often undermined these processes, consistent with prior sarcoma evidence [75, 76]. These findings suggest that adaptation was not solely an individual process, but one shaped by relational and healthcare contexts across the illness trajectory. For adults with sarcoma, adaptation appeared to depend not only on personal coping preferences and resilience, but also on the availability of social support, continuity of care, and access to sarcoma‐informed healthcare professionals.

Finally, participants consistently reported unmet needs across the sarcoma trajectory (Theme 5), particularly regarding timely diagnosis and access to specialist, integrated care that supports recovery, gaps repeatedly highlighted in previous reviews [6, 12]. Early diagnostic experiences shaped subsequent care, with specialist sarcoma support fostering trust, while fragmented diagnostic experiences reinforced uncertainty and abandonment, consistent with evidence that diagnostic delays can carry enduring psychosocial consequences in sarcoma [76]. Many participants also felt ill‐prepared for recovery, reporting limited information about functional consequences and poor access to sarcoma‐informed rehabilitation. Rehabilitation in sarcoma is inherently complex and protracted, shaped by tumour heterogeneity, treatment type, and competing clinical priorities [77]. These findings highlight the importance of coordinated, sarcoma‐centred care to support longer‐term adaptation and quality of life. Moreover, these unmet needs help explain why adaptation was more difficult for some participants than others.

### 4.1 Clinical Implications

Findings from this synthesis indicate that adults with sarcoma may experience substantial psychosocial disruption linked to visible bodily change, reduced function, altered identity, and difficulty adjusting to life after treatment. If adaptation is understood as an ongoing process unfolding across the sarcoma trajectory, these findings highlight the need for assessment and support that extend beyond acute treatment and attend not only to emotional distress, but also to functional, relational, and identity‐related consequences. The findings also support the need for sarcoma‐informed, individualised care including early rehabilitation planning and prehabilitation where appropriate, to prepare individuals for anticipated functional change and reduce long‐term burden [77, 78].

Across the reviewed studies, clear and ongoing communication with sarcoma‐informed professionals was described as central to psychosocial wellbeing and adaptation, highlighting the importance of continuity and sensitivity across the care trajectory. However, given the rarity of sarcoma and the limited number of specialist centres, access to specialist care is often constrained. The findings therefore point not only to the value of specialist input where available, but also to the importance of improving sarcoma awareness among GPs, primary care clinicians, and non‐specialist teams to support earlier recognition, appropriate referral, and more informed care. The pervasive uncertainty described across diagnosis, treatment, and survivorship also suggests a need for psychosocial support that extends beyond acute treatment. Although sarcoma‐specific intervention evidence remains limited, approaches that support meaning‐making, acceptance, and psychological flexibility may be especially relevant for addressing uncertainty, identity disruption, emotional distress, and fear of recurrence within coordinated sarcoma care. In this regard, emerging sarcoma‐specific evidence [71] suggest that greater psychological flexibility is associated with lower fear of recurrence, highlighting its potential value as a therapeutic target in supporting adjustment and wellbeing.

### 4.2 Strengths and Limitations

A key strength of this review is that it synthesised a wide and contemporary body of qualitative research to provide a coherent account of adult psychosocial experiences across the sarcoma trajectory, with most included studies rated as high quality. Analytic depth was strengthened through reflexive team interpretation drawing on expertise in sarcoma care, psycho‐oncology, and lived experience. The present synthesis also extends on prior syntheses findings [11, 12] by focusing exclusively on adults, incorporating recently published studies, and conceptualising adaptation as an ongoing process across the illness trajectory. Rather than treating psychological, social, and functional impact as separate domains, this synthesis highlights how these experiences interact within a broader psychosocial adjustment process, with continuity of sarcoma‐informed care and rehabilitation emerging as central to adaptation.

However, several limitations should be acknowledged. Many studies offered limited reflection on how researcher position and interaction may have shaped data generation and interpretation. In addition, most studies were conducted in Western countries and within specialist and centralised sarcoma services and predominantly reflected post‐treatment experiences. As such, the findings may underrepresent experiences of individuals receiving care in non‐specialist settings, during earlier diagnostic phases, or in palliative contexts. Additionally, the heterogeneity in sarcoma subtype, stage, and treatment pathways may obscure subgroup‐specific experiences. Finally, excluding GIST and Kaposi sarcoma narrows the generalisability of the findings to the sarcoma groups included in this synthesis, as psychosocial experiences in GIST and Kaposi sarcoma may differ due to their distinct clinical and treatment contexts.

### 4.3 Future Directions

Future research should examine how psychosocial needs in sarcoma evolve over time through longitudinal qualitative designs, with particular attention to active treatment, palliative care, surveillance, and recurrence. Greater representation of older adults, ethnic minority groups, and those treated outside specialist centres is needed to address gaps in the evidence base and potential disparities in care. Finally, research evaluating the implementation of sarcoma‐specific rehabilitation and integrated psycho‐oncology interventions would support translation into practice.

## 5 Conclusion

This review shows that sarcoma involves profound and enduring psychosocial disruption alongside ongoing efforts to adapt. The findings suggest that adaptation is best understood as a dynamic process unfolding across diagnosis, treatment, recovery, and survivorship, rather than as a fixed endpoint. Experiences of uncertainty, embodied change, and identity disruption are shaped by relational and healthcare contexts, highlighting the need for coordinated, specialist care that supports meaning‐making, rehabilitation, and longer‐term psychosocial adaptation.

## Author Contributions

**R. Beghean:** conceptualization, methodology, investigation, data curation, formal analysis, validation, visualization, project administration, writing – original draft, writing – review and editing. **L. O'Driscoll:** conceptualization, methodology, investigation, data curation, formal analysis, validation, writing – review and editing. **E. Ryan:** investigation, data curation, validation. **P. Carty:** investigation, data curation, validation. **F. Larkin:** formal analysis, visualization, writing – review and editing. **F. O'Keeffe:** conceptualization, methodology, supervision, validation, writing – review and editing.

## Funding

The authors have nothing to report.

## Conflicts of Interest

The authors declare no conflicts of interest.

## Supporting information


Supporting Information S1

Supporting Information S2

Supporting Information S3

## Data Availability

Data sharing not applicable to this article as no datasets were generated or analysed during the current study.
