# Supplementary material for: Psychosocial Impact of Sarcoma: Challenges and Adaptation, a Meta‐Synthesis
Source: Psychooncology. 2026 Jul 7;35(7):e70534. doi: 10.1002/pon.70534 (PMC13340199; doi:10.1002/pon.70534)
Supplement: Supplementary file 2 — Supporting Information S2 [file PON-35-e70534-s001.docx]

**Supplementary Material 2**

**Search Strategy**

**CHIP**

| Context | Sarcoma individuals at hospitals, outpatient clinics, communities, their homes |
| --- | --- |
| How | Qualitative methods |
| Issues | Psychosocial impact of sarcoma on the individuals with sarcoma    Psychosocial factors influencing the experience    Psychosocial needs of individuals with sarcoma |
| Population | Individuals with Sarcoma |

Concept 1: Sarcoma

Concept 2: Psychosocial impact

**CINAHL**

| Search 1: relevant subject headings for concept 1 | (MH "Sarcoma") OR (MH "Soft Tissue Neoplasms") OR (MH "Bone Neoplasms+") OR (MH "Neoplasms, Connective and Soft Tissue+") |
| --- | --- |
| Search 2: keywords for concept 1 | Sarcoma OR "Bone cancer" OR "Bone neoplasm*" OR  "Bone tumo*" OR  "Bone sarcoma" OR "Soft tissue cancer OR "Soft tissue neoplasm*"OR  "Soft tissue tumo*" OR "soft tissue sarcoma" OR  "perivascular epithelioid cell tumo*" OR  "PEComa" OR “GIST” OR “solitary fibrous tumour” OR “SFT” OR “Vascular Sarcomas” OR “Malignant peripheral nerve sheath tumours” OR “soft tissue tumo*” |
| Search 3: Combine search 1 and 2 with OR |  |
| Search 4: relevant subject headings for concept 2 | (MH "Support, Psychosocial+") OR (MH "Attitude+") OR (MH "Behavior and Behavior Mechanisms+") OR (MH "Self Concept+") OR (MH "Body Image+") OR (MH "Stress, Psychological+") OR (MH "Behavioral Symptoms+") OR (MH "Fatigue+") OR (MH "Mental Health") OR (MH "Psychological Well-Being") OR (MH "Quality of Life+") OR (MH "Adaptation, Psychological+") OR (MH "Coping+") OR (MH "Defense Mechanisms+") OR (MH "Emotions+") OR (MH "Psychosocial Functioning") OR (MH "Psychology, Social+") OR (MH "Pain+") OR (MM "Survivorship") |
| Search 5: keywords for concept 2 | psychosocial OR psycho-social OR "psychosocial factor$" OR socio-emotional OR psychological-social  OR "psychosocial issue*" "psychosocial impact*" OR "Psychosocial implication*" OR "Psychosocial harm*" OR "Psychosocial benefit*" OR Stress OR Anxiety OR Depression OR coping OR "emotional distress" OR resilience OR fatigue OR loneliness OR "sexual function" OR "social support" OR relationships OR "financial stress" OR "money concern" OR "money worry" OR "social isolation" OR "Return to work" OR "return to sport" OR "Self-esteem" OR "body image" OR "Social stigma" OR "emotional adjustment" OR "psychological well-being" OR "adaptive behavio$" OR "patient* experience*" OR survivor* |
| Search 6: Combine search 4 and 5 with OR |  |
| Search 7: Combine search 3 with search 6 with AND |  |
| Limiters: | "interview*" or "focus group*" or "survey*" or "questionnaire*" or "observ*" or "discourse analysis" or "content analysis" or "interpretive phenomenological analysis" or "IPA" or "thematic analysis" or "narrative analysis" or "conversation analysis" or "grounded theory" or "qualitative analysis" or "qualitative" or "mixed method*" |

**Medline**

| Search 1: relevant subject headings for concept 1 | exp "Neoplasms, Connective and Soft Tissue"/ OR exp Sarcoma/ OR exp Bone Neoplasms/ OR exp Soft Tissue Neoplasms |
| --- | --- |
| Search 2: keywords for concept 1 | Sarcoma.mp OR "Bone cancer".mp OR "Bone neoplasm*".mp OR  "Bone tumo*".mp OR  "Bone sarcoma".mp OR "Soft tissue cancer".mp OR "Soft tissue neoplasm*".mp OR  "Soft tissue tumo*".mp OR "soft tissue sarcoma".mp OR  "perivascular epithelioid cell tumo*".mp OR  "PEComa".mp OR “GIST”.mp OR “solitary fibrous tumour”.mp OR “SFT”.mp OR “Vascular Sarcomas”.mp OR “Malignant peripheral nerve sheath tumours”.mp OR “soft tissue tumo*”.mp |
| Search 3: Combine search 1 and 2 with OR |  |
| Search 4: relevant subject headings for concept 2 | exp "Behavior and Behavior Mechanisms"/ OR exp Self Concept/ OR exp Stress, Psychological/ OR exp Behavioral Symptoms/ OR exp Fatigue/ OR exp Mental Health/ OR exp Adaptation, Psychological/ OR exp Defense Mechanisms/ OR exp Emotions/ OR exp Psychosocial Functioning/ OR exp Psychology, Social/ OR exp Pain/ OR Resilience, Psychological/ OR exp "Quality of Life" |
| Search 5: keywords for concept 2 | psychosocial.mp OR psycho-social.mp OR "psychosocial factor$".mp OR socio-emotional.mp OR "psychological-social".mp  OR fatigue.mp OR "psychosocial issue*".mp OR "psychosocial impact*".mp OR "psychosocial implication*".mp OR "psychosocial harm*".mp OR "psychosocial benefit*".mp OR "Social Isolation".mp OR "Social Stigma".mp OR stress.mp OR anxiety.mp OR coping.mp OR "emotional distress".mp OR "sexual function".mp OR "social support".mp OR relationships.mp OR "money concern".mp OR "money worry".mp OR "financial stress".mp OR "return to work".mp OR "return to sport".mp OR loneliness.mp OR "Emotional Adjustment".mp OR "Body Image".mp OR Depression.mp OR "Mental Fatigue".mp OR "Psychological Well-Being".mp OR "Self-esteem".mp OR "adaptive behavio$".mp OR "patient* experience*".mp OR survivor*.mp |
| Search 6: Combine search 4 and 5 with OR |  |
| Search 7: Combine search 3 with search 6 with AND |  |
| Limiters:    Adults  English language  Humans | interview*.mp or "focus group*".mp or survey*.mp or questionnaire*.mp or observ*.mp or "discourse analysis".mp or "content analysis".mp or "interpretive phenomenological analysis".mp or IPA.mp or "thematic analysis".mp or "narrative analysis".mp or "conversation analysis".mp or "grounded theory".mp or "qualitative analysis".mp or qualitative.mp or "mixed method*".mp |

**Web of Science (no subject headings)**

| Search 1: keywords for concept 1 | Sarcoma OR "Bone cancer" OR "Bone neoplasm*" OR  "Bone tumo*" OR  "Bone sarcoma" OR "Soft tissue cancer " OR "Soft tissue neoplasm*"OR  "Soft tissue tumo*" OR "soft tissue sarcoma" OR  "perivascular epithelioid cell tumo*" OR  "PEComa" OR “GIST” OR “solitary fibrous tumour” OR “SFT” OR “Vascular Sarcomas” OR “Malignant peripheral nerve sheath tumours” OR “soft tissue tumo*” |
| --- | --- |
| Search 2: keywords for concept 2 | psychosocial OR psycho-social OR "psychosocial factor$" OR socio-emotional OR psychological-social OR "psychosocial issue*" "psychosocial impact*" OR "Psychosocial implication*" OR "Psychosocial harm*" OR "Psychosocial benefit*" OR emotions OR Stress OR Anxiety OR Depression OR coping OR "emotional distress" OR "cognitive functioning" OR "psychological adaptation" OR "social adaptation" OR resilience OR adjustment OR fatigue OR loneliness OR "sexual function" OR "social support" OR relationships OR "financial stress" OR "money concern" OR "money worry" OR "social isolation" OR "Return to work" OR "return to sport" OR "Self-esteem" OR "Self-concept" OR "body image" OR "Social stigma" OR "emotional adjustment" OR "quality of life" OR "psychological well-being" OR "adaptive behavio$" OR "patient* experience*" OR survivor* |
| Search 3: combine searches with AND |  |
| Search 4: Add limiters with AND | "interview*" or "focus group*" or "survey*" or "questionnaire*" or "observ*" or "discourse analysis" or "content analysis" or "interpretive phenomenological analysis" or "IPA" or "thematic analysis" or "narrative analysis" or "conversation analysis" or "grounded theory" or "qualitative analysis" or "qualitative" or "mixed method*" |

**PsychINFO**

| Search 1: relevant subject headings for concept 1  No relevant subject headings for sarcoma | N/A |
| --- | --- |
| Search 2: keywords for concept 1 | Sarcoma OR "Bone cancer" OR "Bone neoplasm*" OR  "Bone tumo*" OR  "Bone sarcoma" OR "Soft tissue cancer" OR "Soft tissue neoplasm*" OR  "Soft tissue tumo*" OR "soft tissue sarcoma" OR  "perivascular epithelioid cell tumo*" OR  "PEComa" OR “GIST” OR “solitary fibrous tumour” OR “SFT” OR “Vascular Sarcomas” OR “Malignant peripheral nerve sheath tumours” OR “soft tissue tumo*” |
| Search 3: Combine search 1 and 2 with OR |  |
| Search 4: relevant subject headings for concept 2 | (((((((((((DE "Psychosocial Factors" OR DE "Community Resources" OR DE "Protective Factors" OR DE "Psychosocial Outcomes" OR DE "Risk Factors" OR DE "Social Resources")  OR  (MM "Resilience (Psychological)"))  OR  (MM "Psychological Stress"))  OR  (MM "Anxiety"))  OR  (MM "Depression (Emotion)"))  OR  (DE "Coping Behavior" OR DE "Coping Style"))  OR  (MM "Adaptive Behavior"))  OR  (MM "Interpersonal Relationships"))  OR  (DE "Social Support" OR DE "Perceived Social Support"))  OR  (DE "Self-Concept"OR DE "Self-Compassion" OR DE "Self-Confidence" OR DE "Self-Esteem"))  OR  (DE "Quality of Life" OR DE "Health Related Quality of Life" OR DE "Quality of Work Life"))  OR  (DE "Body Image" OR DE "Body Dissatisfaction" OR DE "Body Esteem" OR DE "Body Image Disturbances") |
| Search 5: keywords for concept 2 | psychosocial OR psycho-social OR "psychosocial factor$" OR socio-emotional OR "psychological-social" OR fatigue OR "psychosocial issue*" OR "psychosocial impact*" OR "psychosocial implication*" OR "psychosocial harm*" OR "psychosocial benefit*" OR "Social Isolation" OR "Social Stigma" OR stress OR anxiety OR "adaptive behavio$" OR coping OR "emotional distress" OR "cognitive functioning" OR "psychological adaptation" OR "social adaptation" OR resilience OR adjustment OR "sexual function" OR "social support" OR relationships OR "money concern" OR "money worry" OR "financial stress" OR "return to work" OR "return to sport" OR Resilience, Psychological OR loneliness OR "Interpersonal Relations " OR Emotions OR "Emotional Adjustment" OR Pain OR "Self-Concept" OR "Body Image" OR Depression OR "Mental Fatigue" OR "Quality of Life" OR "Psychological Well-Being" OR "Self-esteem" OR "patient* experience*" OR survivor* |
| Search 6: Combine search 4 and 5 with OR |  |
| Search 7: Combine search 3 with search 6 with AND |  |
| Limiters: | "interview*" or "focus group*" or "survey*" or "questionnaire*" or "observ*" or "discourse analysis" or "content analysis" or "interpretive phenomenological analysis" or "IPA" or "thematic analysis" or "narrative analysis" or "conversation analysis" or "grounded theory" or "qualitative analysis" or "qualitative" or "mixed method*" |
