# Supplementary material for: Psychosocial Impact of Sarcoma: Challenges and Adaptation, a Meta‐Synthesis
Source: Psychooncology. 2026 Jul 7;35(7):e70534. doi: 10.1002/pon.70534 (PMC13340199; doi:10.1002/pon.70534)
Supplement: Supplementary file 3 — Supporting Information S3 [file PON-35-e70534-s003.docx]

# Supplementary Material 3

# Eligibility Criteria

## Inclusion Criteria

- Peer-reviewed empirical studies employing established qualitative methodologies (e.g., interviews, focus groups, qualitative analysis).
- Participants currently or previously diagnosed with sarcoma.
- All sarcoma subtypes and stages, with the exception of Gastrointestinal Stromal Tumours (GIST) and Kaposi’s sarcoma.
- Mixed-methods studies, provided that qualitative data could be clearly extracted and analysed independently.
- Studies published in the English language.
- Participants aged 18 years or older at the time of study participation. Studies including participants under 18 years were eligible only where adult data were analysed and reported separately.
- Studies reporting empirical qualitative findings.
- Studies in which the psychosocial impact of sarcoma was not the primary focus were included where a distinct section, theme, or subtheme addressed psychosocial experiences; only relevant qualitative data were extracted.
- Studies exploring the perspectives of caregivers, family members, or healthcare professionals were included where these accounts explicitly described or illuminated the experiences of individuals living with sarcoma.
- Studies conducted in any geographical location.
- No restrictions were placed on year of publication.

## Exclusion Criteria

- Quantitative studies without a qualitative component.
- Unpublished manuscripts, theses, conference abstracts, editorials, commentaries, or review articles.
- Studies including mixed diagnostic groups (e.g., multiple cancer types) where findings for participants with sarcoma could not be clearly distinguished.
- Studies including mixed respondent groups (e.g., patients and caregivers) where patient data could not be clearly separated.
- Studies focusing exclusively on caregiver, family member, or healthcare professional experiences without direct reference to patient experiences.
- Qualitative studies that did not include raw patient data, such as verbatim quotations.
